# *Trichoderma polysporum* selectively inhibits white-nose syndrome fungal pathogen *Pseudogymnoascus destructans* amidst soil microbes

**DOI:** 10.1186/s40168-018-0512-6

**Published:** 2018-08-08

**Authors:** Amanpreet Singh, Erica Lasek-Nesselquist, Vishnu Chaturvedi, Sudha Chaturvedi

**Affiliations:** 1 Mycology Laboratory, Wadsworth Center, New York State Department of Health, 120 New Scotland Avenue, Albany, NY 12208 USA; 2 Bioinformatics Core, Wadsworth Center, New York State Department of Health, Albany, NY USA; 3 Department of Biomedical Sciences, School of Public Health, University at Albany, Albany, NY USA

**Keywords:** White-nose syndrome, *Pseudogymnoascus destructans*, *Trichoderma polysporum*, Biological decontamination, Biocontrol agent, Selective inhibition, Bat hibernacula, Native soil microbiota, Metagenomics

## Abstract

**Background:**

*Pseudogymnoascus destructans* (*Pd*), the causative fungal agent of white-nose syndrome (WNS), has led to the deaths of millions of hibernating bats in the United States of America (USA) and Canada. Efficient strategies are needed to decontaminate *Pd* from the bat hibernacula to interrupt the disease transmission cycle without affecting the native microbes. Previously, we discovered a novel *Trichoderma polysporum* (*Tp*) strain (WPM 39143), which inhibited the growth of *Pd* in autoclaved soil samples. In the present investigation, we used culture-based approaches to determine *Tp*-induced killing of native and enriched *Pd* in the natural soil of two bat hibernacula. We also assessed the impact of *Tp* treatment on native microbial communities by metagenomics.

**Results:**

Our results demonstrated that *Tp* at the concentration of 10^5^ conidia/g soil caused 100% killing of native *Pd* in culture within 5 weeks of incubation. A 10-fold higher concentration of *Tp* (10^6^ conidia/g soil) killed an enriched *Pd* population (10^5^ conidia/g soil). The 12,507 fungal operational taxonomic units (OTUs, dominated by *Ascomycota* and *Basidiomycota*) and 27,427 bacterial OTUs (dominated by *Acidobacteria* and *Proteobacteria*) comprised the native soil microbes of the two bat hibernacula. No significant differences in fungal and bacterial relative abundances were observed between untreated and *Tp*-treated soil (10^5^ *Tp* conidia/g soil, *p* ≤ 0.05).

**Conclusions:**

Our results suggest that *Tp*-induced killing of *Pd* is highly specific, with minimal to no impact on the indigenous microbes present in the soil samples. These findings provide the scientific rationale for the field trials of *Tp* in the WNS-affected hibernacula for the effective decontamination of *Pd* and the control of WNS.

**Electronic supplementary material:**

The online version of this article (10.1186/s40168-018-0512-6) contains supplementary material, which is available to authorized users.

## Background

*Pseudogymnoascus destructans* (*Pd*), the etiological agent of white-nose syndrome (WNS), has caused significant reductions in hibernating bat populations across the USA and Canada [1–6]. Mortality was first observed in hibernating bats in Howes Cave near Albany, New York (NY), in 2006, and has since spread extensively to 32 US states and 7 Canadian provinces [7]. Recently *Pd* was detected in a little brown bat (*Myotis lucifugus*) as far away as Washington State [8]. Several species of bats are already threatened with extinction, including *Myotis lucifugus* (little brown bat), *Myotis sodalis* (Indiana bat), and *Myotis septentrionalis* (northern long-eared bat) [3, 9]. *Pseudogymnoascus destructans*, a psychrophilic fungus, is well adapted to grow in the cold conditions prevailing in caves and mines [2, 10]. It has been shown to secrete proteolytic enzymes similar to the fungi that cause skin infections (dermatophytes); [2] and has a clonal population in the USA [11–13]. Low body temperature, along with reduced immune system in hibernating bats, provides the optimal growth environment for *Pd* [14–17]. Alarmingly, *Pd* has been found to survive in the affected hibernacula, even in the absence of bats [18–20]. Thus, an infected hibernaculum could remain contaminated with *Pd* for prolonged periods of time and serve as the foci for new infections [21]. Mathematical models have predicted that reducing *Pd* in caves and mines may prevent WNS-associated bat mortality [20, 22]. Currently, efforts are being devoted to the development and testing of chemical and biological agents for the effective eradication of *Pd* from bat hibernacula and hibernating bats. Although these control strategies appear to be promising, they are not being used for the large-scale decontamination of hibernacula, because of the likely off-target effects on the native microbial communities [23–27]. Considering the mass mortality of bats caused by WNS and the economic loss of 22.9 billion dollars to agricultural pest control in the USA annually [28], imminent steps are needed to decontaminate *Pd* from bat hibernacula and break its transmission cycle.

Previously, we characterized a novel, psychrotolerant *Trichoderma polysporum* (*Tp*) strain (WPM 39143) from the William Preserve Mine, Ulster County, NY, one of the mines at the epicenter of the WNS zoonotic [29]. *Tp* grew well at low temperatures of 6–15 °C and inhibited *Pd* in laboratory media and autoclaved soil samples [29]. The present study aimed at further evaluation of *Tp* as an effective biocontrol agent against *Pd*. Specifically, we examined (a) if *Tp* could act as an effective biocontrol agent against *Pd* in the natural soil from bat hibernacula and (b) whether *Tp* treatment impacted the native microbial communities in the natural soil from bat hibernacula.

## Methods

### Fungal strains and media

*Pseudogymnoascus destructans* (*Pd*) strain M1379 and *Tp* strain WPM 39143 were used as described previously [29, 30]. All fungal isolates were maintained on Sabouraud dextrose agar (SDA) slants at 4 °C and stored in 20% glycerol at − 70 °C in sterile cryogenic vials. Sabouraud dextrose agar fortified with an enhanced panel of antibacterials (SDA-A; Additional file 1), SDA-A with cycloheximide (0.2 g/L), and rose bengal agar with chloramphenicol (RBC; 100 μg/ml) were used for the isolation of *Pd*, *Tp*, and other fungi from bat hibernacula as described previously [30]. Potato dextrose agar (PDA) and water agar (WA) were used to induce spore formation in *Pd* and *Tp*. Millet seeds extract was used to fortify nutrients in the soil samples from bat hibernacula for *Tp* and *Pd* interaction studies. In brief, 50 g of millet seeds was added in 250 ml water, was autoclaved at 121 °C for 20 min, and was mixed with an additional 250 ml sterilized water. The resulting extract was passed through muslin cloth, autoclaved as above, allowed to cool, and stored at 4 °C until used. All the experiments were performed in the biosafety cabinet in a biosafety level 2 laboratory.

### Biocontrol application of *Tp* in natural soil

To determine *Tp*-induced killing of *Pd* in a natural soil, one soil sample from Aeolus cave (AC), Bennington County, VT (dark black in color; collection date of 11/13/2015), and one soil sample from Barton Hill Mine (BHM), Essex County, NY (course sediments; collection date of 12/28/2015), were used. Soil samples, approximately 100 g, from each site were weighed, transferred to an autoclaved mortar and pestle, and mixed gently to obtain a homogeneous mixture. The mixture was aliquoted into eight vials for each site, with each vial containing 5 g of soil. Four vials were inoculated with *Pd* (10^5^ conidia/g soil), and the remaining four vials received sterilized water. Following 1-week post-incubation, two of the four *Pd*-containing vials received *Tp* (10^5^ conidia/g soil) to obtain a 1:1 ratio of *Pd* to *Tp*. The other two *Pd* containing vials received sterilized water (*Pd* only). Of the remaining four vials in which *Pd* was not inoculated, two received *Tp* (10^5^ conidia/g soil), which served as *Tp* only controls, while the remaining two vials received sterilized water and served as soil only controls (Additional file 2). All the vials were incubated at 10 °C for 5 weeks.

To determine the number of *Tp* required for killing *Pd* in subsequent experiments, 10-fold (10^6^ conidia/g soil) and 100-fold (10^7^ conidia/g soil) higher concentrations of *Tp* conidia than *Pd* conidia (10^5^ conidia/g soil) were added and these soil samples were incubated at 10 °C and then processed at 1, 3, and 5 weeks post-incubation (Additional file 3).

### Culture recovery of *Pd*, *Tp*, and other fungi

Following incubation of soil samples at 10 °C for 1–5 weeks, 100 mg of soil sample was removed from each vial in duplicate and transferred into 2-ml screw cap vials. The soil sample was suspended in 500 μl of sterilized water and vortexed vigorously. Ten-fold dilutions of the supernatant were prepared, and 50 μl of each dilution was plated onto various media plates (150 mm diameter) in duplicate. The plates were incubated at 10 °C and checked periodically for the recovery of *Pd*, *Tp*, and other fungi as described previously [30]. In brief, SDA-A medium was used to determine the *Tp* colony-forming units (CFUs), SDA-A with cycloheximide was used to determine *Pd* CFU, and the RBC medium was used for the determination of other fungi present in the cave and mine soil samples. The percent killing of *Pd* by *Tp* was calculated using a formula 1 − (*Pd* CFU experiment/*Pd* CFU control) × 100.

### Dual culture challenge studies

For dual culture challenge studies, approximately 1 × 1 mm of freshly grown *Tp* fungal hyphal mat was inoculated on one side of the SDA plate. Similarly, freshly grown fungal hyphal mat from various fungi obtained from both AC and BHM were inoculated on the opposite side of the plate. The interactions between *Tp* and other fungal isolates were assessed by measuring colony diameter following 18 days post-incubation at 10 °C. The *Tp* and different fungal isolate cultures alone served as a control.

Statistical analysis for culture-based assays (CFU enumeration and colony diameter) was performed using GraphPad Prism software (GraphPad, San Diego, CA, USA). The comparison of two groups was performed using a two-tailed unpaired *t*-test with a *p* value of ≤ 0.05 accepted as significant.

### Metagenomics of soil samples

#### DNA libraries

For the microbial community analysis, DNA from 100 mg of untreated and *Tp*-treated (10^5^ and 10^6^ conidia/g soil for AC and 10^5^ conidia/g soil for BHM) soil samples (in duplicate) were extracted with the Powersoil DNA isolation kit (MO BIO Laboratories, Carlsbad, CA, USA) (Additional file 4). DNA concentrations were measured using a Nanodrop spectrophotometer ND 2000 (Nano-Drop Technologies, Wilmington, DE, USA). DNA libraries were prepared using a two-step PCR. In the first PCR, the primer sets targeted the ITS2 region of the ribosomal RNA (rRNA) gene of fungi [31] and the hypervariable region V4 of the 16S rRNA gene of bacteria and archaea [32]. The same Illumina adaptor sequences were added to both fungal and bacterial/archaeal primers. Thus, the primer set used for the first PCR for fungi had the following sequence: Forward primer 5′- TCGTCGGCAGCGTCAGATGTGTATAAGAGACAG**AACTTTYRRCAAYGGATCWCT-3′** (locus specific sequence in bold) and Reverse primer 5′- GTCTCGTGGGCTCGGAGATGTGTATAAGAGACAG**AGCCTCCGCTTATTGATATGCTTAART** -3′ (locus-specific sequence in bold). The primer set used for the first PCR for bacteria/archaea had the following sequence: Forward primer 5′- TCGTCGGCAGCGTCAGATGTGTATAAGAGACAG**GTGYCAGCMGCCGCGGTAA-3′** (locus-specific sequence in bold) and Reverse primer 5′- GTCTCGTGGGCTCGGAGATGTGTATAAGAGACAG**GGACTACNVGGGTWTCTAAT-3′** (locus-specific sequence in bold). PCR reactions were carried out in a total volume of 25 μl. Three microliters of extracted DNA (5 ng/μl) was added to the PCR reaction containing 15.1 μl of sterile water, 0.2 μl of bovine serum albumin, 2.5 μl of Accutaq LA buffer, 2 μl of dNTPs, 0.2 μl of Accutaq™ LA DNA polymerase (Sigma-Aldrich, St. Louis, MO, USA), and 1 μl each of fungal and bacterial/archaeal forward and reverse primers. All the PCR reactions were carried out in triplicate on a C1000 Touch Thermal Cycler (BioRad, Hercules, CA, USA). The thermocycling conditions for fungal-specific PCR were initial denaturation at 95 °C for 1 min, followed by 27 cycles of denaturation at 94 °C for 30 s, annealing at 55 °C for 1 min, extension at 68 °C for 1 min, followed by final extension at 68 °C for 5 min. For bacterial/archaeal-specific PCR, thermocycling conditions were 95 °C for 1 min, followed by 27 cycles of denaturation at 94 °C for 30 s, annealing at 63 °C for 1 min, extension at 68 °C for 1 min, and a final extension at 68 °C for 5 min. The first-stage PCR products were run on the agarose gel to confirm the length of the respective bands. Triplicate samples were pooled to limit possible PCR artifacts. An Agencourt AMPure XP PCR purification kit (Beckman Coulter, Inc., Indianapolis, IN, USA) was used for the purification of the PCR products. The second PCR step added dual indices (barcode) along with Illumina sequencing adaptors using the Nextera XT index kit (Illumina-16S Metagenomics protocol). PCR reactions were carried out in a total volume of 50 μl, which contained 5 μl of PCR product from the first PCR and the 2× KAPA Hifi HotStart Ready mix. The reaction conditions were initial denaturation at 95 °C for 3 min, followed by eight cycles of denaturation at 95 °C for 30 s, annealing at 55 °C for 30 s, extension at 72 °C for 30 s, and a final extension at 72 °C for 5 min. All PCR reactions were performed in triplicate. The PCR products were cleaned using an Agencourt AMPure XP PCR purification kit. DNA concentration was measured using a Qubit 2.0 Fluorometer (Invitrogen, Carlsbad, CA, USA) and the size was measured with an Agilent Tapestation using the D1000 High Sensitivity kit. The resulting DNA libraries were pooled, denatured, and sequenced on MiSeq using MiSeq Reagent kit v3 (600 cycles) (Illumina Inc., San Diego, CA, USA) by the Wadsworth Center Advanced Sequencing Core Facility.

#### Sequence processing

The Wadsworth Sequencing Core demultiplexed reads and removed primer and linker sequences before analysis. The subroutines of BBTools v36.38 [33], BBmerge and BBduk, merged all pairs of forward and reverse reads, quality trimmed merged pairs (with the parameters trimq = 20, minq = 20, minlength = 150, minavgquality = 20, efilter = 3, mininsert = 250, mininsert0 = 250), and removed any remaining forward and reverse primers for fungal and bacterial samples. The ITS2 region for taxonomic identification was extracted from fungal sequences using ITSx v.1.0.11 [34], which removed 5.8S and 28S regions from merged sequences. Chimeric sequences were removed using USearch61 with a modified Unite “ITS2-only” reference dataset [35] (version 7.2 release 28.06.2017) which was designed to serve as a chimera-free reference database. Chimeric 16S sequences were removed from bacterial samples in QIIME v.1.9.0 [36] via Usearch61 [37, 38] and the most recent version of the Greengenes database (gg_13_8 minor release of gg_13_5 from 15.08.2013). Using default parameters, QIIME further processed quality-filtered sequences, which were then clustered into operational taxonomic units (OTUs) at 97% similarity by the UCLUST algorithm [37] and assigned taxonomy by mothur v.1.25 [39, 40] with the pick open reference OTU option and a default confidence level of 0.5. The latest releases of the Greengenes 16s (gg_13_8) and UNITE datasets (version 7 release 28.06.2017) [41], both clustered at 97% similarity, served as the reference databases for clustering and taxonomic assignment of bacterial and fungal sequences, respectively.

#### Sample statistics

For bacterial and fungal datasets, Chao1, observed species, and Simpson’s index estimates of alpha diversity were calculated in QIIME with rarefied OTU tables. Significant differences across treatments and locations were assessed via a non-parametric *t*-test with 999 permutations of the *p* value. QIIME also calculated beta diversity with Bray Curtis, and Euclidean metrics and significant differences in community composition were identified with an analysis of similarities (ANOSIM). Principal component analysis plots were generated in R v.3.3.0 [42] with Euclidean distance matrices to confirm consistency among replicates and verify that most of the variation in datasets was derived from differences in treatment and location (data not shown).

Bacterial and fungal samples were rarefied to depths of 190,000 and 92,226, respectively, and relative abundances were summarized to a genus level (if possible). Abundance count at the phylum and genus levels were exported from QIIME and analyzed in DESeq2 v.3.5 [43] in R to identify significant changes in taxonomic composition. Only taxa with four or more counts across samples were included in DESeq2 analyses to remove sparse OTUs.

### Fungal identification

All fungi recovered from the soil samples were identified by morphological and molecular methods [44]. For molecular testing, DNA from pure fungal colonies was extracted using MasterPureTM Complete DNA and RNA purification kits (Epicenter, Madison, WI, USA) as per manufacturer’s instructions. The extracted DNA was used for the amplification of internal transcribed spacer (ITS) regions 1 and 2 (ITS1, 5.8S, and ITS2) of the ribosomal gene as described previously [30]. In some instances, where the ITS region failed to provide fungal identification, the D1/D2 region of the large subunit (LSU) of the 28S rDNA gene was amplified [30]. PCR was carried out as described in White et al. [45]. PCR products were cleaned with ExoSAP-IT (USB Corp., Cleveland, OH, USA) and sequenced at the Wadsworth Center Advanced Genomics Core. The sequences were assembled and edited for accuracy using Sequencher software 4.8 (Gen Codes Corp., Ann Arbor, MI, USA). All unknown sequences were compared to the NCBI GenBank database with blast [46] and the Westerdijk Fungal Biodiversity Institute database [47] for fungal identifications; % identity of ≥ 97 was used for species confirmation. In case of discrepant results between the two databases, the Westerdijk Fungal Biodiversity Institute database was preferred as it has curated sequences.

## Results

### Biocontrol of *Pd* in natural soil – A culture-based approach

**Fig. 1:**
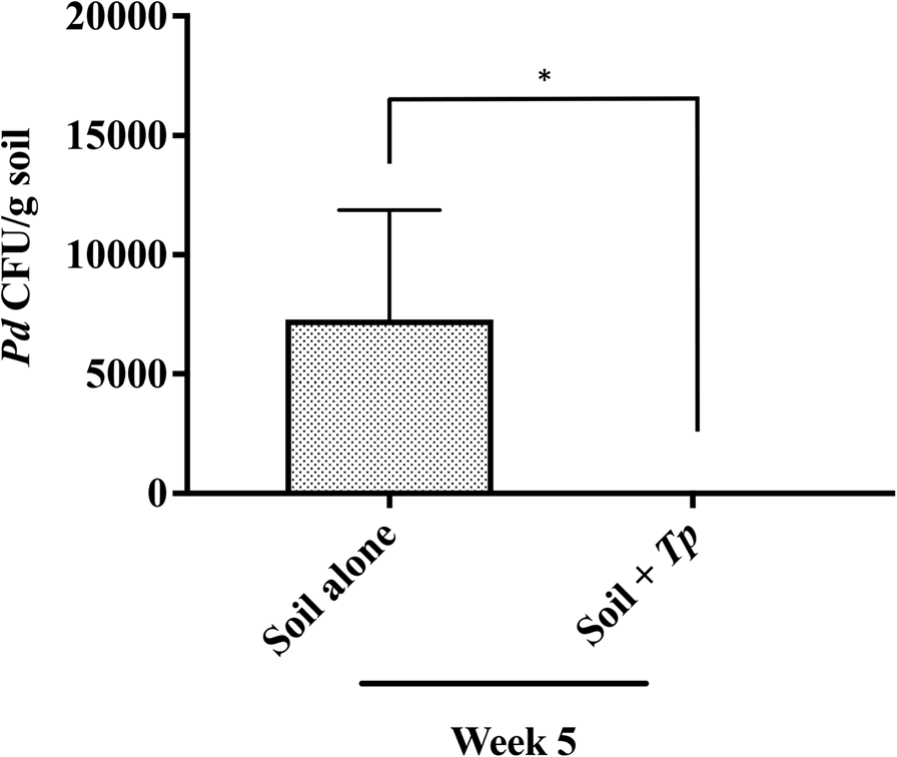
Inhibition of native *Pd* by *Tp* in AC soil samples. Untreated soil (soil alone) and soil treated with *Tp* (10^5^ conidia/g soil) were incubated at 10 °C. Five weeks post-incubation, two aliquots from each sample were processed for the recovery of *Pd*. *Tp* induced 100% killing of native *Pd* in the AC soil samples (*p* < 0.05)

**Fig. 2:**
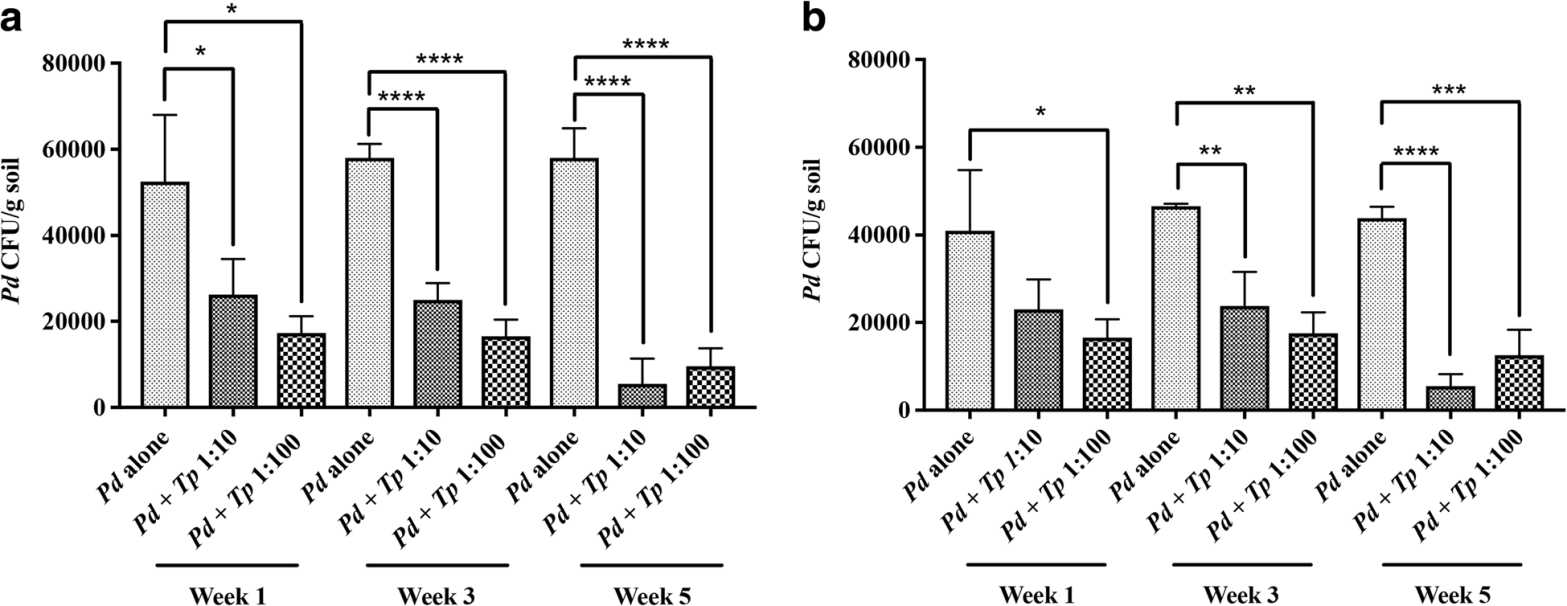
Inhibition of enriched *Pd* by *Tp* in AC soil samples. **a** Soil samples from AC were inoculated with *Pd* (10^5^ conidia/g soil) in duplicate. Following 7 days post-incubation at 10 °C, *Tp* at 10-fold (10^6^ conidia/g soil) or 100-fold (10^7^ conidia/g soil) to that of *Pd* (10^5^ conidia/g soil) was added. Untreated soil (soil alone) and soil containing *Tp* served as controls. Two aliquots from each sample were processed for the recovery of *Pd* in culture at week 1, 3, and 5 post-incubation. Approximately, 50 and 67% killing of *Pd* was observed with 10-fold and 100-fold higher *Tp* at week 1 post-incubation, which gradually increased to 57 and 72% at week 3 post-incubation and 95 and 84% at week 5 post-incubation. **b** Soil samples were inoculated with *Pd* and treated with *Tp* as explained in **a**, except that these samples also received 1% millet seeds extract. Addition of millet seeds extract did not enhance *Tp* killing of *Pd*.

*Pd* was recovered from native AC soil at ~ 10^4^ CFU/g soil (Fig. 1). *Pd* recovery was very low from BHM (~ 8 CFU/g soil), consistent with our previous observations [30]. Treatment of AC soil with *Tp* (10^5^ conidia/g soil) resulted in 100% inhibition of the native *Pd* population within 5 weeks post-incubation at 10 °C, confirming high biocontrol potential of *Tp* (Fig. 1). However, when AC and BHM soil samples were enriched with *Pd* (10^5^ conidia/g soil), the *Tp*-induced killing of *Pd* was markedly reduced to approximately 40 to 43% (data not shown). Maximum killing of *Pd* in enriched soil was observed, when *Tp* inoculum was increased from 1-fold (10^5^conidia/g soil) to 10-fold (10^6^ conidia/g soil) to that of *Pd* (10^5^ conidia/g soil). Although *Tp* killed *Pd* as early as 1-week post-incubation, 95% killing was achievable at 5-week post-incubation (Fig. 2a). Further increase in *Tp* to 100-fold of *Pd* was not effective as the killing of *Pd* was observed to be only 84% at 5-week post-incubation (Fig. 2a). Enriching soil nutrients by adding millet seeds extract did not impact *Tp*-induced killing of *Pd* as only 87 and 72% *Pd* was killed with the additions of 10-fold and 100-fold more *Tp*, respectively (Fig. 2b). These results indicated that the ratio of *Tp* to *Pd* of 10:1 appeared to optimally render the maximum inhibitory effect. The soil microbial communities did not hamper *Tp* growth in any of the soil samples tested. The *Tp* growth was not only sustained but also increased with prolonged incubation (data not shown).

### Effect of *Tp* treatment on microbial communities of cave and mine soil samples—a metagenomics approach

#### Sequence analysis

A total of 2,003,427 fungal sequences passed initial quality filters. Of these, 96% (1,921,639) passed QIIME’s quality standards as well, while 0.07% (1347) were flagged as chimeric and removed (Table 1). This resulted in an average of 192,163 sequences per sample (*N* = 10), which were clustered into 12,507 OTUs. For all bacterial samples, a total of 3,295,648 sequences passed the initial quality filters of BBtools with 97% of these sequences (3,177,499) accepted by QIIME and 0.6% (21,503) identified as chimeric and removed (Table 1). On average, there were 317,749 sequences per sample (*N* = 10), which clustered into 27,427 OTUs.

**Table 1:** Fungal and bacterial community analyses by high throughput sequencing

Sample	Fungi		Bacteria	
	BBmerge and bbduk	QIIME and Usearch61	BBmerge and bbduk	QIIME and Usearch61
AC Soil alone 1	96,993	92,225	247,465	237,561
AC Soil alone 2	161,501	153,529	321,874	308,610
AC Soil + *Tp* A1	214,333	204,336	393,209	377,754
AC Soil + *Tp* A2	166,355	158,564	344,568	330,694
AC Soil + *Tp* B1	323,672	312,063	444,201	427,840
AC Soil + *Tp* B2	116,711	105,622	207,965	199,979
BH Soil alone 1	156,003	150,631	275,597	267,273
BH Soil alone 2	233,462	225,964	306,924	298,202
BH Soil + *Tp* A1	205,092	200,594	304,892	295,134
BH Soil + *Tp* A2	329,305	318,111	448,953	434,452
Total	2,003,427	1,921,639	3,295,648	3,177,499

Soil samples from Aeolus Cave (AC) and Barton Hill Mine (BHM) were treated with *Tp* at the concentration of 10^5^ conidia/g soil (A1, A2) or 10^6^ conidia/g soil (B1, B2) followed by gDNA extraction, PCR, and high throughput sequencing. Untreated soil samples (soil alone) were included for comparison. Note, due to small number of *Pd* recovered from BHM soil, the *Tp* treatment was limited to 10^5^ conidia/g soil

#### *Tp* treatment and location effects on alpha and beta diversity of soil samples

No significant differences in the alpha diversity were observed across treatments or geographical locations for either bacterial or fungal communities (Additional file 5). Fungal community composition changes, as assessed by the beta diversity estimates, also did not differ significantly due to *Tp* treatment of either AC or BHM soils. However, there was a significant difference in community composition between the two locations (ANOSIM test statistic = 1.0, *p* value 0.003). Similarly, bacterial beta diversity was not significantly altered by *Tp* treatment within AC or BHM but varied significantly between sites.

#### Treatment effect on the abundance of fungal and bacterial communities at the phylum and genus levels

**Fig. 3:**
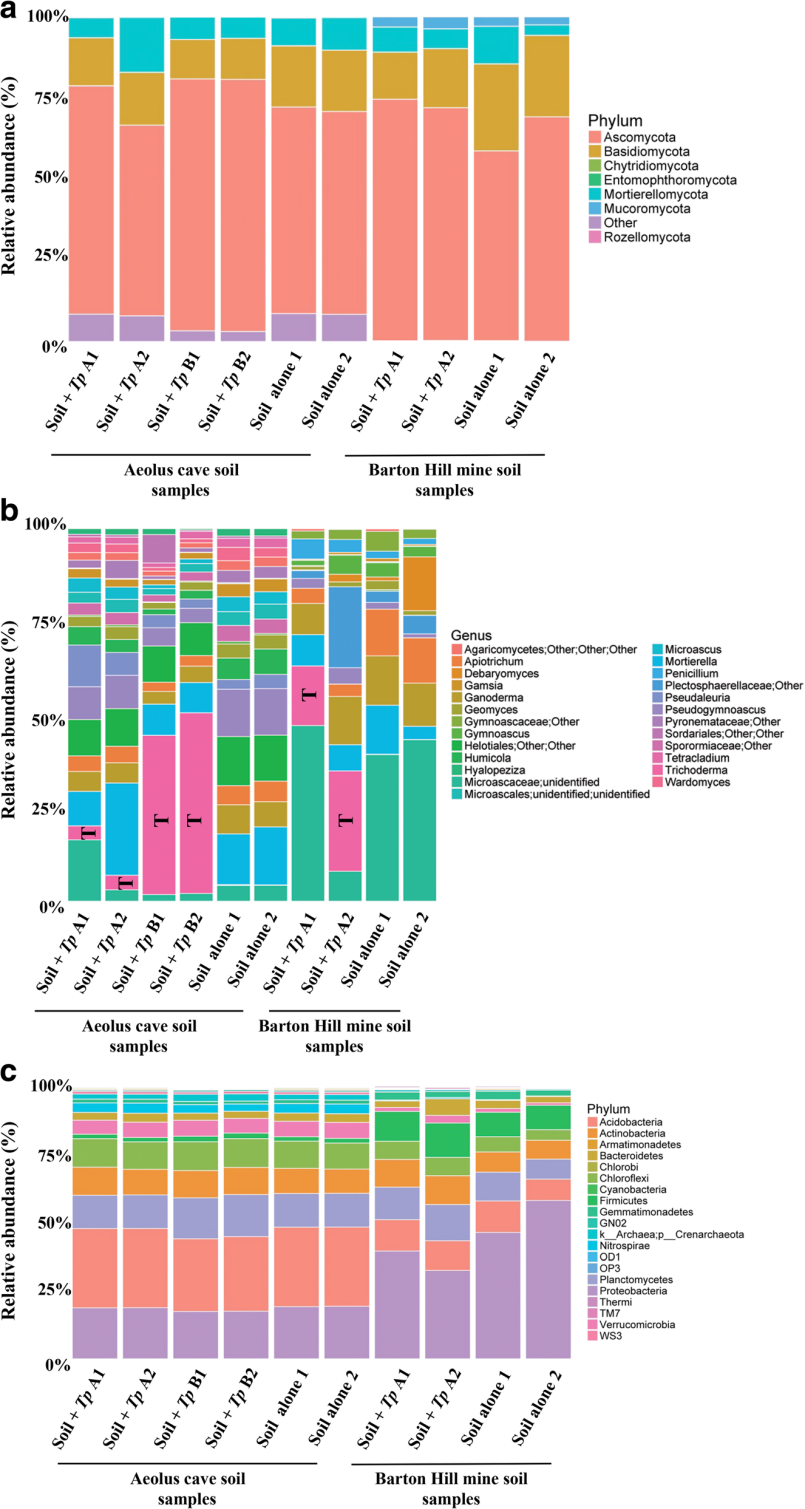
Relative abundance of fungal and bacterial communities in soil samples from bat hibernacula with or without *Tp* treatment. The soil samples from Aeolus Cave (AC) and Barton Hill Mine (BHM) were treated with *Tp* at the concentration of 10^5^ conidia/g soil (A1 & A2) or 10^6^ conidia/g soil (B1 & B2). Untreated soil samples (soil alone) were included for comparison. gDNA was extracted followed by PCR and high throughput sequencing. **a** The relative abundance of fungal phyla is shown. *Ascomycota* dominated, followed by *Basidiomycota* and early diverging fungal lineages (*Chytridiomycota*, *Entomophthoromycota*, *Mortierellomycota*, *Mucormycota*, and *Rozellomycota*) from both AC and BHM. **b** The relative distribution of 25 most abundant fungal genera is shown. The increases in *Trichoderma* as represented by “T” in both AC and BHM were due to the exogenous addition of *Tp*. **c** The relative abundance of bacterial phyla is shown. *Acidobacteria* dominated AC soil and *Protobacteria* dominated BHM soil

For fungi, 97% of all sequences could be assigned at the phylum level, with seven phyla identified from AC and five phyla identified from BHM. Both AC and BHM were dominated by OTUs from *Ascomycota* and *Basidiomycota*, which comprised over 80% of the fungal communities (Fig. 3a). Soil subjected to *Tp* treatment (10^5^ conidia/g soil) showed no significant differences in the phylum abundances for fungi at either AC or BHM (Additional file 6). However, there was a significant increase in *Ascomycota* for AC soil subjected to a higher *Tp* treatment (10^6^ conidia/g soil, Additional file 6), which reflected the dramatic increase in *Trichoderma* in these samples (Fig. 3b). Similarly, analysis at the genus level revealed highly significant increases in *Trichoderma* for both AC and BHM for both treatments, with few other taxa being affected (Fig. 3b, Additional file 7). Although no significant changes in *Pseudogymnoascus* abundance were observed (Additional file 7), the genus encompasses several species, which might not be influenced by *Tp* treatment.

For bacteria/archaea, a total of 50 phyla were identified from AC, whereas only 37 were identified from BHM with 0.27% of the sequences designated as unclassified (Bacteria; Other). The five most abundant phyla in AC were *Acidobacteria* (28%), *Proteobacteria* (18%), *Planctomycetes* (13%), *Chloroflexi* (10%), and *Actinobacteria* (9.5%) (Fig. 3c). The most abundant phyla identified from BHM included *Planctomycetes* (11%), *Acidobacteria* (10%), *Firmicutes* (10%), and *Actinobacteria* (8.8%), with *Proteobacteria* (44%) comprising almost half of the BHM bacterial community (Fig. 3c). Despite apparent differences in the community richness and evenness between the AC and BHM, there were no significant differences in alpha diversity (Additional file 5). There were also no significant differences in bacterial relative abundances between untreated and *Tp*-treated soil (10^5^ *Tp* conidia/g soil) for AC at the phylum or genus level (Additional files 8 and 9). For AC soil samples treated with 10^6^ *Tp* conidia/g soil, 12 phyla and 40 genera were significantly affected compared to untreated soil samples (Additional files 8 and 9). For BHM soil samples treated with 10^5^ *Tp* conidia/g soil, no phyla and only 12 genera showed significant changes (Additional files 8 and 9).

### *Tp* interaction with other cave/mine fungi—a culture-based approach

**Fig. 4:**
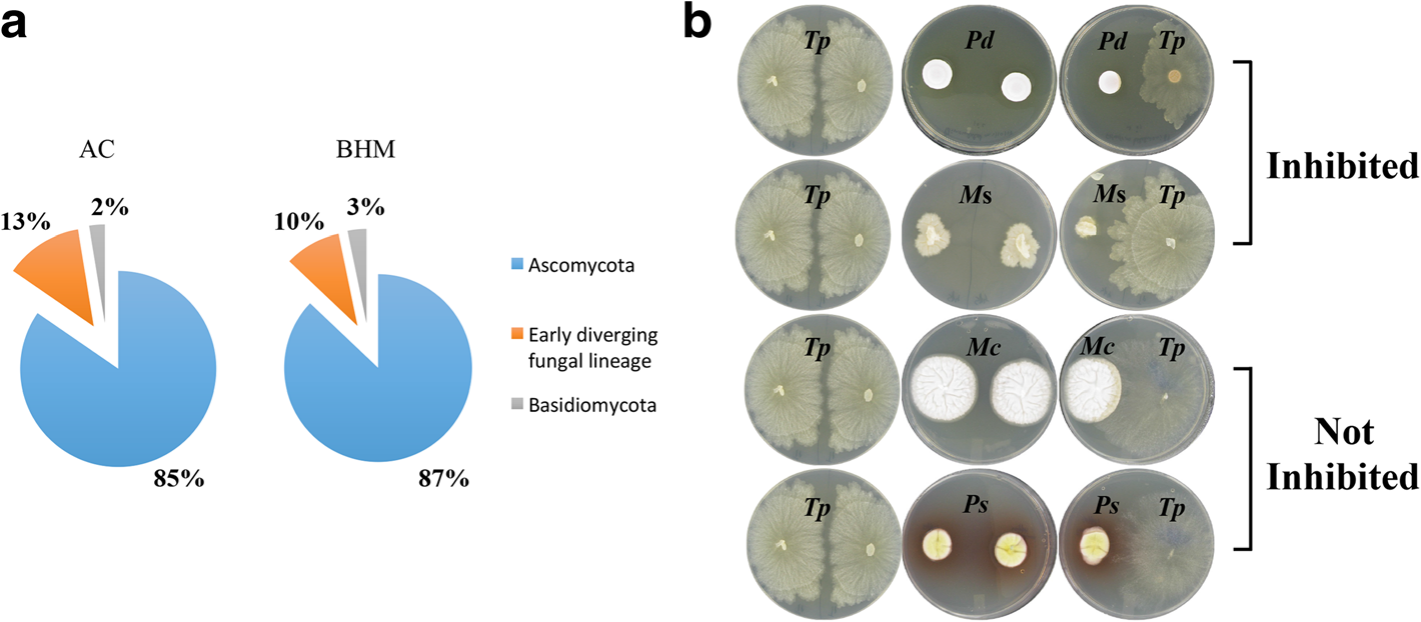
Fungal recovery from bat hibernacula and their interaction with *Tp*. **a** Soil samples from Aeolus Cave (AC) and Barton Hill Mine (BHM) were suspended in sterile water and then spread on culture media plates. Colonies recovered were identified by sequencing of the ITS and D1/D2 regions of the ribosomal gene followed by BLAST search. Pie charts represent the relative distributions of fungal phyla. *Ascomycota* dominated followed by Early Diverging Fungal Lineage, and *Basidiomycota* in both AC and BHM. **b** Interaction of *Tp* with other fungal species isolated from AC and BHM was carried out on SDA plate and the results of these interactions were assessed 18 days post-incubation at 10 °C. Upper panel denotes *Tp*-induced inhibition of *Pd* and *Microascus* species (*M*s). Lower panel denotes fungal species not inhibited by *Tp*. Two such examples are *Mortierella clonocystis* (*Mc*) and *Penicillium sopii* (*Ps*)

A total of 39 fungal species were recovered from AC, and of these, 85% belonged to *Ascomycota*, 13% belonged to early diverging fungal lineage (EDFL), and 2% belonged to *Basidiomycota* (Fig. 4a, Additional file 10). Of the 31 fungal species recovered from BHM, 87% belonged to *Ascomycota*, 10% belonged to EDFL, and 3% belonged to *Basidiomycota* (Fig. 4a, Additional file 11). Thus, *Ascomycota* dominated the fungal species followed by EDFL and *Basidiomycota* from both AC and BHM.

#### Dual challenge study

The dual interaction studies indicated that *Tp* is highly specific in inhibiting *Pd*. In addition to *Pd*, only one fungal isolate belonging to genus *Microascus* was inhibited. The rest of the 67 fungal species collectively identified from both AC and BHM were not inhibited, including the closely related species *Pseudogymnoascus pannorum* (Fig. 4b, Additional files 10 and 11).

## Discussion

We demonstrated that the biocontrol agent *Tp* inhibited *Pd* in the presence of microbes that are native to the soil from the affected hibernacula. This finding further expanded our earlier observations of the efficacy of *Tp* in killing of *Pd* in autoclaved soil samples [29]. Another important finding of this study was the specificity of *Tp* for killing *Pd* with minimal to no impact on the microbial diversity and community structures of soil samples tested from both AC and BHM. Even though the microbial community compositions of AC and BHM were significantly different, they were not affected by *Tp* treatment, suggesting that the soil communities are relatively robust and indifferent to *Tp* treatment. The combined culture-based and metagenomics approaches allowed us to follow the fate of the biocontrol agent and its target in the treated soil. Culture-based monitoring of *Pd* and *Tp* was important to estimate loss of viable organisms while DNA-based approaches provided better census of microbial communities.

Several DNA and culture-based studies have revealed wide distribution and persistence of *Pd* in bat hibernacula [18, 21, 48, 49]. Other published cave fungal surveys indicate that *Pd* could survive and persist in bat hibernacula for prolonged periods and can have impact on both WNS disease management and epidemiology [18]. To eradicate *Pd* from bat hibernacula, we need highly competent biocontrol agent, which can grow, sustain, and selectively kill *Pd* without impacting the hibernacula ecosystem. The *Tp* strain used in this investigation fulfills all these criteria, thereby strengthening the argument for application of *Tp* as a potential biocontrol agent against *Pd* in caves and mines.

In concordance with metagenomics analysis, the dual-culture challenge studies of *Tp* with several fungi recovered from AC and BHM revealed that except for one isolate of *Microascus* species, the growth of other fungi was not affected by *Tp*. *Microascus* is a soil saprophyte and a common agent of bio-deterioration [50]. Since other species in the genus *Microascus* were not inhibited by *Tp*, we do not anticipate deleterious effects on cave/mine ecosystem. Conversely, *Tp* grew well in the presence of both fast- and slow-growing fungi, as well as in the presence of other microbial communities indigenous to the soil samples tested. The high survival potential of *Tp* in hibernacula soil suggests its ability to survive under unfavorable conditions and high reproductive capacity.

Microbial communities play several critical roles in the soil, including organic matter decomposition and control of its cycle, regulation of mineral nutrient availability, and nitrogen fixation [51]. Thousands of bacterial, archaeal, and eukaryotic organisms are present in natural soil and collectively contribute to maintaining the myriad of functions of soil. Microbial inoculation of a biocontrol agent can cause tremendous changes in the number and composition of taxonomic groups. These changes can increase the diversity of the soil samples while also having toxic effects on the indigenous microbes [52]. Thus, the practical use of any microbial inoculation should be rigorously tested in a laboratory setting to avoid any deleterious effects to microbial diversity in soils. To this end, we have rigorously tested the use of *Tp* as a biocontrol agent for the eradication of *Pd* in cave and mine soil samples and *Tp* treatment in large had no impact on the native microbial communities other than *Pd*.

## Conclusions

The present study demonstrates the remarkable specificity and high potency of *Tp* for killing *Pd* in the presence of indigenous microbial communities with minimal to no impact on the microbial community structure or diversity present in AC and BHM soil samples. The study rigorously tested the application of *Tp* in natural soil samples in a lab setting, with results that strengthens the argument for *Tp*’s application as a biocontrol agent under field conditions.

## Additional files

Additional file 1: Concentrations of extended panel of antibiotics in Sabouraud dextrose agar. (DOCX 13 kb)

Additional file 2: Flow chart of *Pd* and *Tp* (1:1 ratio) interaction in AC and BHM soil samples. (TIF 294 kb)

Additional file 3: Flow chart of *Pd* and *Tp* (1:10 and 1:100 ratio) interaction in AC soil samples. (TIF 324 kb)

Additional file 4: Flow chart of *Pd* and *Tp* interaction (1:1 and 1:10 ratio) in AC soil and *Pd* and *Tp* interaction (1:1 ratio) in BHM soil followed by DNA extraction and metagenomics analysis. (TIF 184 kb)

Additional file 5: Comparison of alpha-diversity among *Tp* treatments and locations using a non-parametric *t*-test. (XLSX 55 kb)

Additional file 6: DESeq2 analysis of changes in the abundance of fungal phyla in soil from bat hibernacula with or without *Tp* treatment. (XLSX 55 kb)

Additional file 7: DESeq2 analysis of changes in the abundance of fungal genera in soil from bat hibernacula with or without *Tp* treatment. (XLSX 97 kb)

Additional file 8: DESeq2 analysis of changes in the abundance of bacterial phyla in soil from bat hibernacula with or without *Tp* treatment. (XLSX 19 kb)

Additional file 9: DESeq2 analysis of changes in abundance of bacterial genera in soil from bat hibernacula with or without *Tp* treatment. (XLSX 193 kb)

Additional file 10: Fungi recovered from AC soil and assessed for growth inhibition by *Tp*. (DOCX 20 kb)

Additional file 11: Fungi recovered from BHM soil and assessed for growth inhibition by *Tp*. (DOCX 17 kb)

## Abbreviations

AC: Aeolus cave
ANOSIM: Analysis of similarities
BHM: Barton Hill Mine
CFU: Colony-forming unit
EDFL: Early diverging fungal lineage
ITS: Internal transcribed spacer
LSU: Large subunit
OTUs: Operational taxonomic units
Pd: *Pseudogymnoascus destructans*
PDA: Potato dextrose agar
RBC: Rose bengal agar with chloramphenicol
rRNA: Ribosomal RNA
SDA: Sabouraud dextrose agar
*Tp*: *Trichoderma polysporum*
WA: Water agar
WNS: White-nose syndrome
