# Supplementary material for: Trichoderma polysporum selectively inhibits white-nose syndrome fungal pathogen Pseudogymnoascus destructans amidst soil microbes
Source: Microbiome. 2018 Aug 8;6:139. doi: 10.1186/s40168-018-0512-6 (PMC6083572; doi:10.1186/s40168-018-0512-6)
Supplement: Supplementary file 1 — Concentrations of extended panel of antibiotics in Sabouraud dextrose agar. (DOCX 13 kb) [file 40168_2018_512_MOESM1_ESM.docx]

**Additional file 8. Concentrations of extended panel of antibiotics in Sabouraud dextroase agar**

| **Number** | **Antibiotic** | **Concentration used (µg/ml)** |
| --- | --- | --- |
| 1. | Amikacin sulfate salt | 150 |
| 2. | Ampicillin sodium salt | 40 |
| 3. | Carbenicillin disodium salt | 122 |
| 4. | Keflex | 40 |
| 4. | Colistin sulfonate sodium salt | 32 |
| 5. | Kanamycin | 40 |
| 6. | Ofloxacin | 80 |
| 7. | Tetracycline | 40 |
| 8. | Gentamicin sulfate salt | 70 |
| 9. | Chloramphenicol | 25 |
| 10. | Vancomycin HCl | 43 |
| 11. | Penicillin/Streptomycin | 20 U/ml / 40 U/ml |
