# Supplementary figures and images for: Trichoderma polysporum selectively inhibits white-nose syndrome fungal pathogen Pseudogymnoascus destructans amidst soil microbes
Source: Microbiome. 2018 Aug 8;6:139. doi: 10.1186/s40168-018-0512-6 (PMC6083572; doi:10.1186/s40168-018-0512-6)

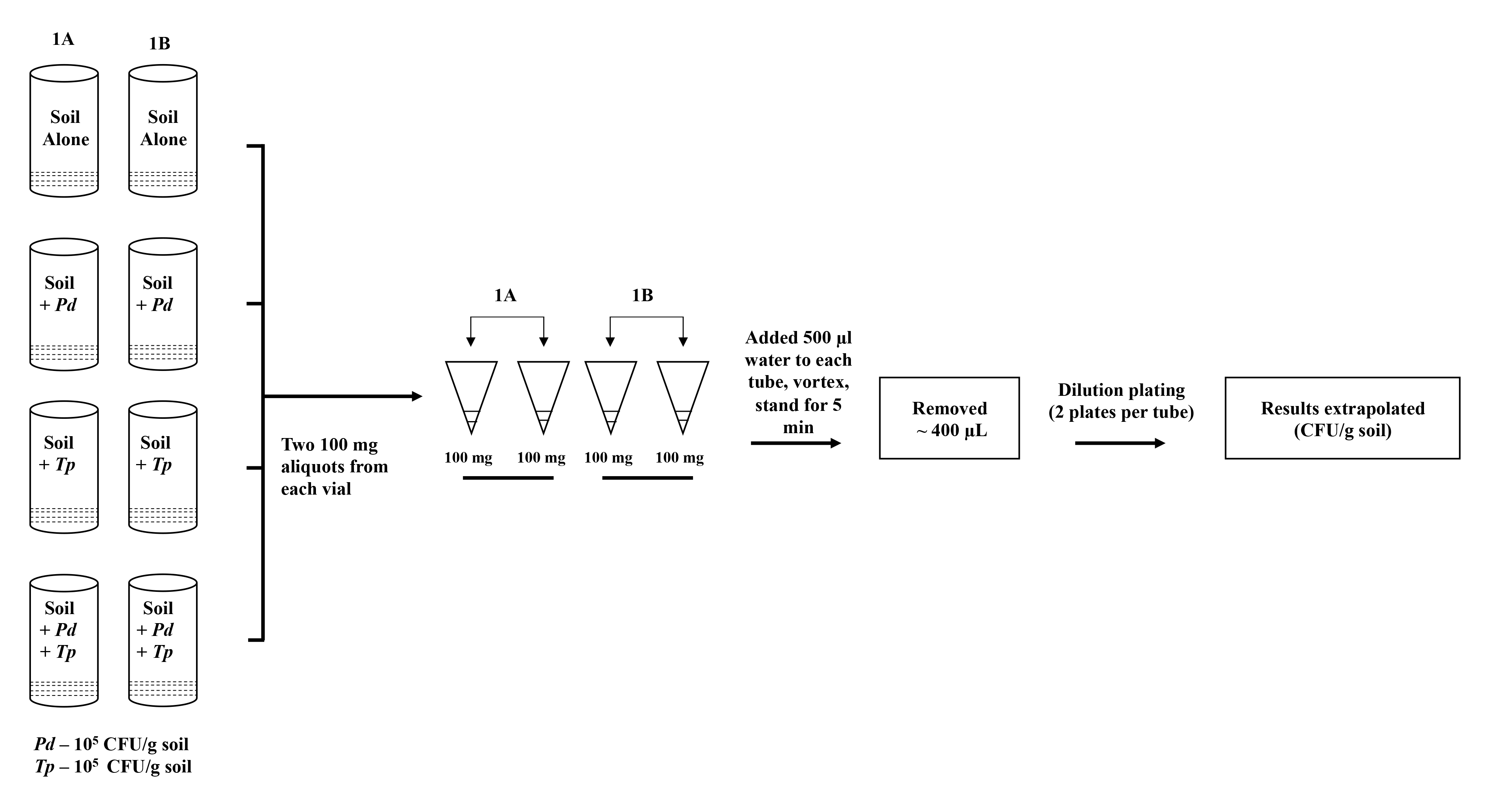

Supplement: Supplementary file 2 — Flow chart of Pd and Tp (1:1 ratio) interaction in AC and BHM soil samples. (TIF 294 kb) [file 40168_2018_512_MOESM2_ESM.tif]

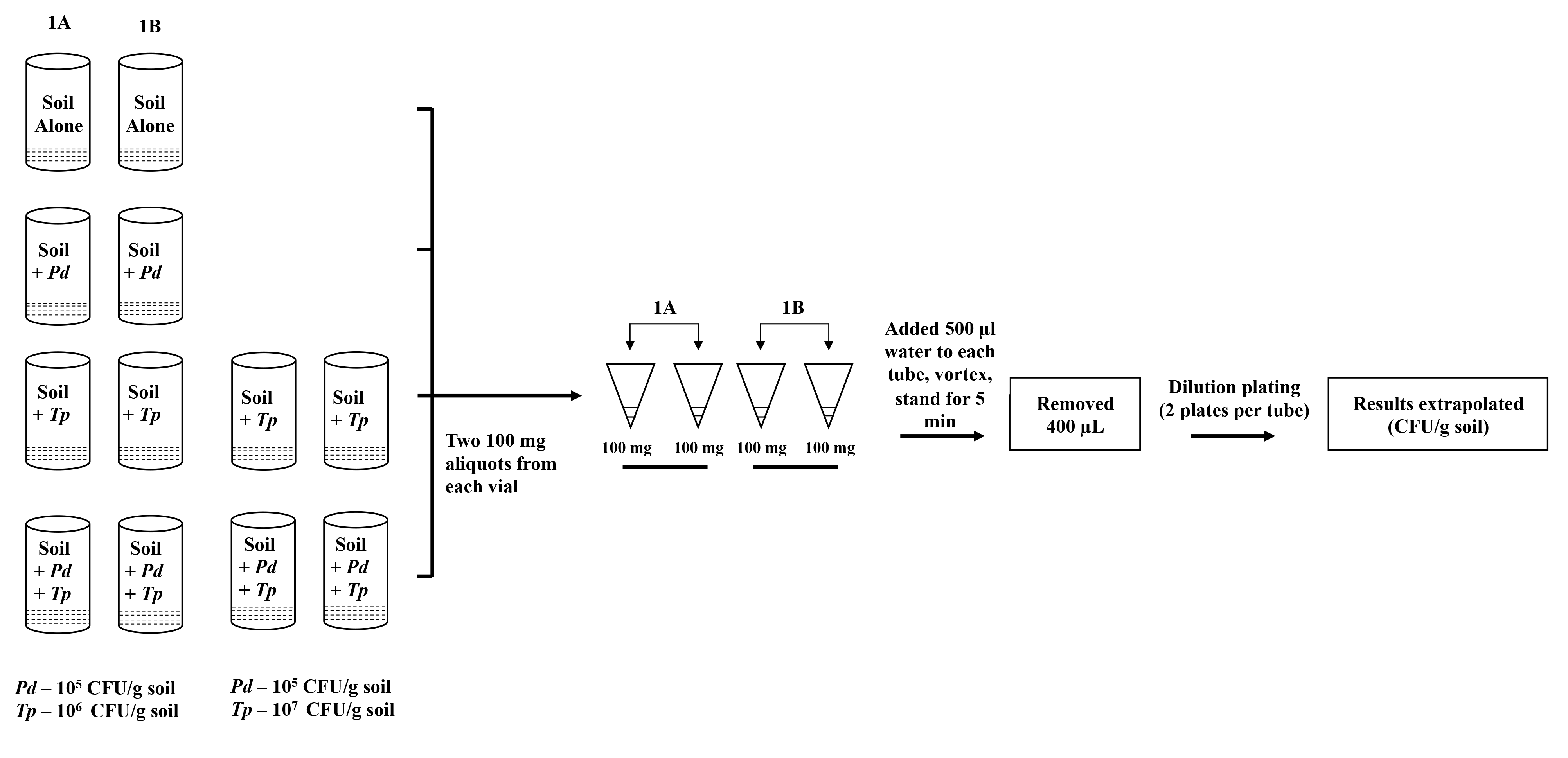

Supplement: Supplementary file 3 — Flow chart of Pd and Tp (1:10 and 1:100 ratio) interaction in AC soil samples. (TIF 324 kb) [file 40168_2018_512_MOESM3_ESM.tif]

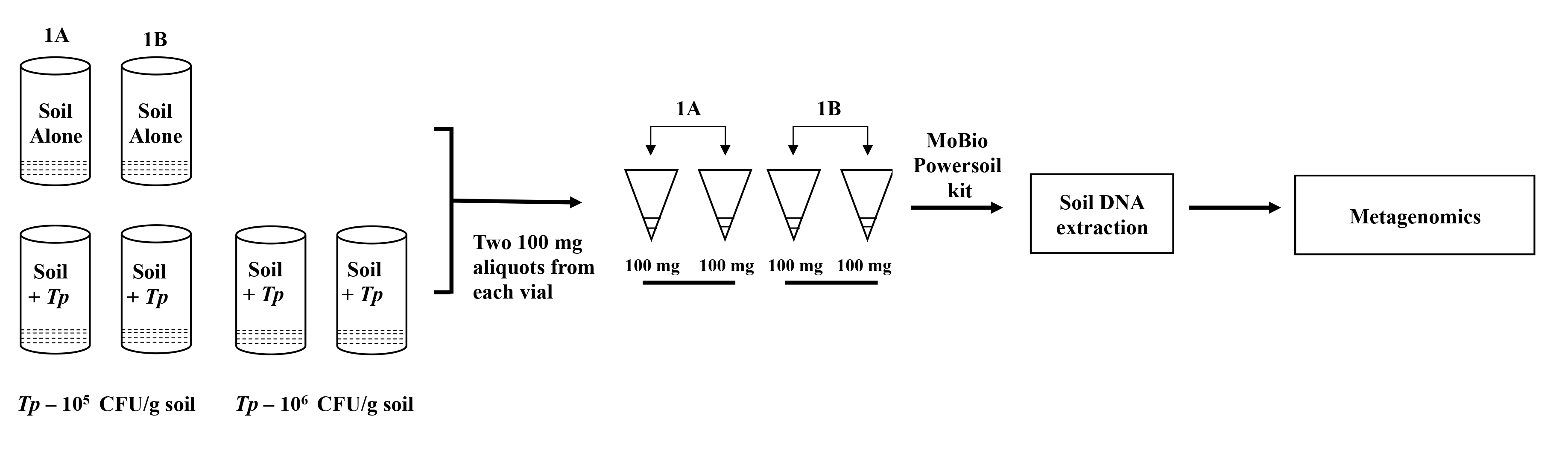

Supplement: Supplementary file 4 — Flow chart of Pd and Tp interaction (1:1 and 1:10 ratio) in AC soil and Pd and Tp interaction (1:1 ratio) in BHM soil followed by DNA extraction and metagenomics analysis. (TIF 184 kb) [file 40168_2018_512_MOESM4_ESM.tif]
