# Supplementary material for: Trichoderma polysporum selectively inhibits white-nose syndrome fungal pathogen Pseudogymnoascus destructans amidst soil microbes
Source: Microbiome. 2018 Aug 8;6:139. doi: 10.1186/s40168-018-0512-6 (PMC6083572; doi:10.1186/s40168-018-0512-6)
Supplement: Supplementary file 10 — Fungi recovered from AC soil and assessed for growth inhibition by Tp. (DOCX 20 kb) [file 40168_2018_512_MOESM10_ESM.docx]

**Additional file 6. Fungi recovered from AC soil and assessed for growth inhibition by *Tp***

| **No.** | **Fungal Species** | **Accession numbers** | **CFU/g**  **of Soil** | **Phylum** | ***Tp*-induced**  **growth**  **inhibition** |
| --- | --- | --- | --- | --- | --- |
| 1. | *Pseudogymnoascus destructans* | MH481333 | 7500^*^ | Ascomycota | **Yes** |
| 2. | *Trichosporon dulcitum* | MH481319 | 4851 | Basidiomycota | No |
| 3. | *Hormiactis candida* | MH481320 | 1583 | Ascomycota | No |
| 4. | *Microascus caviaformis* | MH481337 | 1563 | Ascomycota | No |
| 5. | *Oidiodendron truncatum* | MH481334 | 1562 | Ascomycota |  |
| 6. | *Microascus albonigrescens* | MH481329 | 1500 | Ascomycota | No |
| 7. | *Wardomyces inflatus* | MH481325 | 1417 | Ascomycota | No |
| 8. | *Trichosporiella cerbriformis* | MH481331 | 1229 | Ascomycota | No |
| 9. | *Mortierella antartica* | MH481327 | 583 | EDFL^**^ | No |
| 10. | *Pseudogymnoascus pannorum* | MH481335 | 583 | Ascomycota | No |
| 11. | *Polypaecilum botryoides* | MH481328 | 375 | Ascomycota | No |
| 12. | *Gymnoascus clone* | MH481339 | 375 | Ascomycota | No |
| 13. | *Scopulariopsis atra* | MH481340 | 375 | Ascomycota | No |
| 14. | *Syctalidium thermophilum* | MH481343 | 375 | Ascomycota | No |
| 15. | *Mortierella rostafinskii* | MH481342 | 313 | EDFL | No |
| 16. | *Doratomyces stemonitis* | MH481326 | 312 | Ascomycota | No |
| 17. | *Mucor hiemalis* | MH481319 | 250 | EDFL | No |
| 18. | *Pseudeurotium sp.* | MH481321 | 250 | Ascomycota | No |
| 19. | *Mortierella gamsii* | MH481322 | 250 | EDFL | No |
| 20. | *Trichoderma viridescens* | MH481323 | 208 | Ascomycota | No |
| 21. | *Microascus species* | MH481338 | 188 | Ascomycota | **Yes** |
| 22. | *Arthroderma cuniculi* | MH481341 | 188 | Ascomycota | No |
| 23. | *Leuconeurospora polypaeciloides* | MH481324 | 167 | Ascomycota | No |
| 24. | *Mortierella clonocystis* | MH481332 | 167 | EDFL | No |
| 25. | *Thelebolus globosus* | MH481330 | 83 | Ascomycota | No |
| 26. | *Isaria javanica* | MH481336 | 83 | Ascomycota | No |
| 27. | *Penicillium chrysogenum* | MH481344 | 63 | Ascomycota | No |
| 28. | *Cosmopora viridescens* | MH481345 | 63 | Ascomycota | No |
| 29. | *Oidiodendron species* | MH481346 | 63 | Ascomycota | No |
| 30. | *Chaetomium species* | MH481347 | 63 | Ascomycota | No |
| 31. | *Pseudogymnoascus species* | MH481348 | 63 | Ascomycota | No |
| 32. | *Chaetomidium leptoderma* | MH481349 | 63 | Ascomycota | No |
| 33. | *Doratomyces nanus* | MH481350 | 63 | Ascomycota | No |
| 34. | *Wardomyces humicola* | MH481351 | 63 | Ascomycota | No |
| 35. | *Wardomyces species* | MH481352 | 63 | Ascomycota | No |
| 36. | *Arthroderma silverae* | MH481353 | 63 | Ascomycota | No |
| 37. | *Arthroderma species* | MH481354 | 63 | Ascomycota | No |
| 38. | *Microascus caviariformis* | MH481355 | 63 | Ascomycota | No |
| 39. | *Trichoderma polysproum* | MH481356 | 63 | Ascomycota | No |

^*^ Relative abundance of fungi in descending order

^**^ Early Diverging Fungal Lineage
