# Supplementary material for: Trichoderma polysporum selectively inhibits white-nose syndrome fungal pathogen Pseudogymnoascus destructans amidst soil microbes
Source: Microbiome. 2018 Aug 8;6:139. doi: 10.1186/s40168-018-0512-6 (PMC6083572; doi:10.1186/s40168-018-0512-6)
Supplement: Supplementary file 11 — Fungi recovered from BHM soil and assessed for growth inhibition by Tp. (DOCX 17 kb) [file 40168_2018_512_MOESM11_ESM.docx]

**Additional file 7. Fungi recovered from BHM soil and assessed for growth inhibition by *Tp***

| **No.** | **Fungal Species** | **Accession**  **numbers** | **CFU/g**  **of Soil** | **Phylum** | ***Tp*-induced**  **growth**  **inhibition** |
| --- | --- | --- | --- | --- | --- |
| 1. | *Penicillium pancosmium* | MH481288 | 1750^*^ | Ascomycota | No |
| 2. | *Trichosporon dulcitum* | MH481314 | 1750 | Basidiomycota | No |
| 3. | *Kernia species* | MH481297 | 1116 | Ascomycota | No |
| 4. | *Pseudogymnoascus pannorum* | MH481293 | 1092 | Ascomycota | No |
| 5. | *Penicillium roseopurpureum* | MH481292 | 1083 | Ascomycota | No |
| 6. | *Mortierella alpina* | MH481290 | 766 | EDFL^**^ | No |
| 7. | *Penicillium ubiquetum* | MH481287 | 667 | Ascomycota | No |
| 8. | *Pseudogymnoascus sp.* | MH481291 | 542 | Ascomycota | No |
| 9. | *Penicillium biorgeianum* | MH481289 | 417 | Ascomycota | No |
| 10. | *Sarocladium strictum* | MH481313 | 375 | Ascomycota | No |
| 11. | *Penicillium concentricum* | MH481312 | 250 | Ascomycota | No |
| 12. | *Westerdykella cylindrical* | MH481315 | 250 | Ascomycota | No |
| 13. | *Pochonia suchlasporia* | MH481311 | 158 | Ascomycota | No |
| 14. | *Penicillium commune* | MH481304 | 142 | Ascomycota | No |
| 15. | *Trichoderma atroviride* | MH481294 | 83 | Ascomycota | No |
| 16. | *Penicillium soppii* | MH481295 | 83 | Ascomycota | No |
| 17. | *Ascomycete sp.* | MH481296 | 83 | Ascomycota | No |
| 18. | *Mortierella gamsii* | MH481304 | 83 | EDFL | No |
| 19. | *Acremonium furcatum* | MH481310 | 75 | Ascomycota | No |
| 20. | *Chrysosporium merdarium* | MH481316 | 63 | Ascomycota | No |
| 21. | *Penicillium janczewskii* | MH481298 | 58 | Ascomycota | No |
| 22. | *Mortierella sp.* | MH481299 | 33 | EDFL | No |
| 23. | *Penicillium brevistipitatum* | MH481308 | 33 | Ascomycota | No |
| 24. | *Eupenicillium catenatum* | MH481300 | 17 | Ascomycota | No |
| 25. | *Doratomyces stemonitis* | MH481301 | 8 | Ascomycota | No |
| 26. | *Oidiodendron truncatum* | MH481302 | 8 | Ascomycota | No |
| 27. | *Microascus sp.* | MH481303 | 8 | Ascomycota | No |
| 28. | *Geomyces species* | MH481305 | 8 | Ascomycota | No |
| 29. | *Pseudogymnoascus destructans* | MH481306 | 8 | Ascomycota | **Yes** |
| 30. | *Polypaecilum botryoides* | MH481309 | 8 | Ascomycota | No |
| 31. | *Trichoderma polysporum* | MH481317 | 8 | Ascomycota | No |

^*^ Relative abundance of fungi in descending order

^**^ Early Diverging Fungal Lineage
